# Switchable Multifunctional Meta-Surface Composed by Dielectric-Metal Hybrid Antenna Array Architecture

**DOI:** 10.3390/nano11112862

**Published:** 2021-10-27

**Authors:** Yule Huang, Jiaxin Yang, Ying Zhang, Zhongchao Wei, Hongzhan Liu, Jianping Guo

**Affiliations:** Guangdong Provincial Key Laboratory of Nanophotonic Functional Materials and Devices, School of Information and Optoelectronic Science and Engineering, South China Normal University, Guangzhou 510006, China; 2019022134@m.scnu.edu.cn (Y.H.); 2020022324@m.scnu.edu.cn (J.Y.); 2020022436@m.scnu.edu.cn (Y.Z.); wzc@scnu.edu.cn (Z.W.)

**Keywords:** optical antenna, perfect absorber, polarization converter, terahertz device

## Abstract

Strontium titanate (STO), the dielectric material, has caught the world’s attention due to its outstanding properties, such as high permittivity, high refractive index, and low loss in the terahertz band. Its permittivity is relevant to the environment temperature. Herein, a multifunctional meta-surface composed of a dielectric-metal hybrid antenna array has been demonstrated, which is a single-layer STO elliptic cylinder. On the one hand, when the environment temperature is 300 K, the proposed meta-surface can achieve perfect absorption and polarization conversion in the frequency range from 0.1 to 0.25 THz; particularly, the meta-surface absorptance can reach 99.97% and 99.92% at a frequency of 0.103 and 0.13 THz respectively, and while it is used as a polarization conversion device, the degree of circular polarization and the ellipticity angle can reach 0.986 and 44.5° at a frequency of 0.228 THz. On the other hand, when the environment temperature changes from 300 to 450 K, the absorption peak changes with the temperature, and the average absorptance reaches 96% at resonance frequency. The proposed meta-surface can be applied in many fields, such as optical sensing, imaging, and energy harvesting. Moreover, it provides a potential solution to research the integrated device in a complex electromagnetic environment.

## 1. Introduction

In recent years, both scientific and engineering applications have been greatly encouraged for their great potential in electromagnetic (EM) wave manipulation [1,2]. Meta-surface, as a two-dimensional (2D) plane structure of metamaterial, can be conveniently used to modulate the amplitude, phase, and polarization of EM waves [3,4]. At present, a relatively novel meta-surface design method is to use algorithm optimization (Particle Swarm Optimization, PSO) to obtain the optimal unit structure to design the required meta-surface. As mentioned in Reference [5], an artificial magnetic conductor with 29% fractional bandwidth was designed at an operating frequency of 12 GHz via the PSO algorithm, and in Reference [6], a second-order bandpass frequency selective surface at 10 GHz with 20% fractional bandwidth was proposed and realized by using the PSO algorithm. There is also a meta-surface design method for directivity improvement, and in Reference [7], an effective technique was come up with for radiation patterns’ improvement of the Fabry–Perot cavity antenna by improving the near-field phase distribution, that led to a 5.6 dBi increase in the peak directivity of the antenna. Additionally, based on the operation method for changing the structure and arrangement of meta-atoms [8], lots of meta-surfaces with different functions have been proposed and designed, such as polarization manipulation [9,10,11], abnormal reflection [12,13], focusing lens [14,15,16], and absorber [17,18,19], etc. Although these meta-surfaces have been designed well, the function of these proposed meta-surfaces is mostly single, which is not suitable for the high-integration electromagnetic system and the complex optical integration system; hence, multifunctional meta-surfaces are proposed and have gradually become a research hotspot. In Reference [20], based on VO_2_ and graphene materials, a multifunctional meta-surface with perfect absorption and polarization conversion functions has been designed by changing the temperature and voltage. In Reference [21], the multifunctional meta-surface composed of VO_2_ and metal has shown anisotropic or isotropic characteristics at different temperatures to realize the functions of perfect absorption and polarization conversion. In References [22,23,24], the proposed meta-surface has achieved many modulation capabilities via adding a circuit system to control the working status of active devices in the structure. However, an array of the mentioned multifunctional meta-surfaces are composed of complex sandwich or multilayer structures, which make the structure complex and the preparation process difficult. Additionally, it is even necessary to change the environment conditions or add the active devices to realize polarization conversion and perfect absorption, which not only increases the complexity of the operation, but also limits the flexibility and applicability of the multifunctional meta-surface.

The optical antenna can localize the electromagnetic (EM) waves in a sub-wavelength structure and realize the conversion between the free space electromagnetic field and the local field by utilizing the characteristics of surface plasmon [25,26,27]. Meta-surface has been widely used in the field of the optical antenna. For example, EM meta-surfaces have recently been successfully used to manipulate the near-field of aperture antennas for a higher gain and beam steering, as explained in the all-metal wideband meta-surface for near-field transformation of medium-to-high gain electromagnetic sources, and also the single-layer polarization-insensitive frequency selective surface for beam reconfigurability of monopole antennas [28,29]. In terms of meta-surface design approach, there are many different design ideas, such as the method of using all-dielectric structures, printed layers, or all-metal [29,30,31]. However, as for the research of optical antennas, it has mainly focused on precious metal materials, such as gold and silver. There is a large intrinsic loss in precious metal materials, which can lead the application of plasmon antennas on optical integration systems to be limited [32,33,34]. High refractive index dielectric materials not only maintain the resonance characteristics, which are similar to metal particles, but also have higher efficiency than metal nanoparticles. This type of material provides a new idea for the design of a low-loss optical antenna [35]. Strontium titanate (STO) has some unique properties in the terahertz band, including high permittivity, high refractive index, low dielectric loss, excellent insulation, and stable chemical stability [36,37]. With such excellent properties, STO may become a new material for designing the optical antenna, and there is another characteristic for STO whereby its permittivity could be dynamically adjusted via changing temperature, which indicates that it may be designed as a temperature-tunable device.

Based on the STO material, we propose a multifunctional meta-surface composed of the dielectric-metal hybrid single-layer antenna array structure that can realize perfect absorption as well as the polarization conversion function in the frequency range from 0.1 to 0.25 THz, without adjusting the environment temperature, changing the structure, or adding active devices. Especially, when the temperature is controlled at 300 K, the absorptance can reach up to 99.97% and 99.92% at 0.103 and 0.13 THz respectively, and the degree of circular polarization and ellipticity angle can reach 0.986 and 44.5° respectively, at the frequency of 0.228 THz. When the temperature changes from 300 to 450 K, the proposed meta-surface can work well as an absorber, and even the average value of absorptance of resonance frequency is still over 96%.

## 2. Materials and Methods

We have mentioned that the STO material has a high refractive index in the terahertz band. Here, we will discuss the complex relative permittivity and refractive index of STO in detail. Firstly, the frequency-dependent complex relative permittivity of STO can be described by [36,37,38]:

(1) $$\varepsilon_{w}=\varepsilon_{\infty }+\frac{f}{w_{0}^{2}-w^{2}-iw\gamma }$$

where $\varepsilon_{\infty }$ = 9.6 is the high-frequency bulk permittivity, *f* = 2.3 × 10^6^ cm^−2^ is a temperature-independent oscillator strength, and ω is the angular frequency of the incident THz wave. In addition, $w_{0}$ and γ are the soft-mode frequency and soft-mode damping factor, which can be respectively written as:

(2) $$w_{0}(T)[cm^{-1}]=\sqrt{31.2(T-42.5)}$$

(3) $$\gamma (T)[cm^{-1}]=-3.3+0.094T$$

where T is the temperature (K).

**Figure 1:**
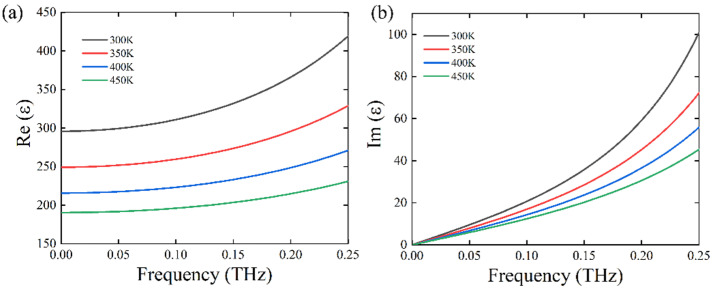
The calculated permittivity of STO under different temperatures. (**a**) Real part of permittivity of STO. (**b**) Imaginary part of permittivity of STO.

**Figure 2:**
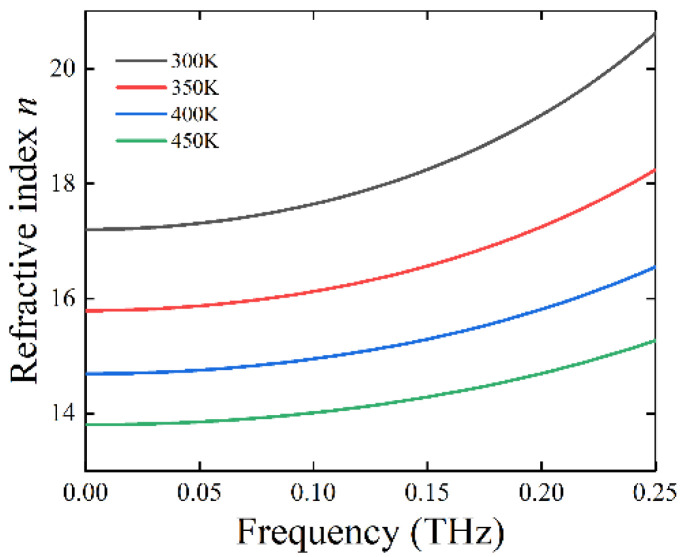
The relative refractive index, *n*, of strontium titanate.

Obviously, the real and imaginary parts of the relative permittivity (Re(*ε*), Im(*ε*)) of STO material are associated with ambient temperature, while the frequency of the incident THz wave has been fixed. As shown in Figure 1a,b, the Re(*ε*) and Im(*ε*) are changeable at a frequency ranging from 0.001 to 0.25 THz when the ambient temperature has been changed from 300 to 450 K. In the lower frequency range, from 0.1 to 0.25 THz, the values of Re(*ε*) and Im(*ε*) have almost changed with the decrease of temperature, which indicates that the complex relative permittivity of STO material is extremely sensitive to the change of ambient temperature, suggesting potential application in the temperature-tunable integrated devices. Meanwhile, the frequency-dependent complex relative refractive index of STO can be described by:

(4) $$n=\sqrt{\varepsilon_{r}\mu_{r}}$$

where *n* is the refractive index, $\varepsilon_{r}$ is the relative permittivity of STO, and μ_r_~1 is the relative permeability for the most nonmagnetic materials. Therefore, the *n* of STO can be obtained under different temperatures, as shown in Figure 2, which indicates that STO is a high refractive index material in the range of 0.001 to 0.25 THz. In Reference [39], it is also proven that the STO is a high refractive index material at the terahertz band, via related experiments.

**Figure 3:**
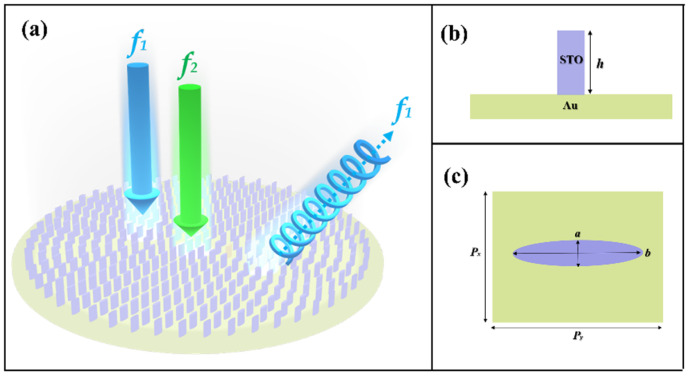
(**a**) The schematic diagram of the designed multifunctional meta-surface composed of the dielectric-metal hybrid single-layer antenna array. (**b**) Side view of the single-layer antenna. (**c**) Top view of the single-layer antenna.

Figure 3 shows the schematic diagram of the designed multi-functional meta-surface consisting of the dielectric-metal hybrid single-layer antenna array. As shown in Figure 3a, the three-dimensional (3D) view of the structure, the meta-surface comprised one layer of the elliptical STO antenna on the metal substrate (Au); furthermore, the two arrows represent linearly polarized lights with different frequencies. What it expresses is when the two linearly polarized lights with different frequencies pass through the meta-surface, one linearly polarized light with frequency *f_1_* is converted into circularly polarized light, and another linearly polarized light with frequency *f_2_* is absorbed. The upper layer of the meta-surface is comprised of the STO elliptical antenna with the same major axis a, minor axis b, and height h. Figure 3b,c present the single-layer antenna structure composed of one STO elliptical antenna on the upper sides of the metal substrate.

When transmitting through an elliptic cylinder structure, the light polarized along the major axis and that along the minor axis both experience phase shifts (the two phase-shifts are assumed as $\varphi_{x}^{\prime }$ and $\varphi_{y}^{\prime }$, respectively). Due to the chromatic dispersion produced by a combination of the material dispersion and the waveguide dispersion, the refractive index for the light polarized along the major axis (assumed as $n_{x}(\omega )$, where ω is the optical frequency) and that for the light polarized along the minor axis (assumed as $n_{y}(\omega )$) both vary with the wavelength of the incident light field [14]. Therefore, the difference between the phase shift of the light linearly polarized along the major axis and that along the minor axis, i.e.,

(5) $$\Delta \varphi_{x,y}=\varphi_{x}^{\prime }-\varphi_{y}^{\prime }=\{n_{x}(\omega )-n_{y}(\omega )\}\frac{\omega }{c}L^{\prime },$$

is wavelength-dependent. In Equation (5), *c* is the light velocity in the vacuum, and *L′* is the propagation distance. Herein, we first designed an antenna structure with polarization conversion and a perfect absorption function under the room temperature *T* = 300 K. In order to find the expected antenna structure, we utilized the three-dimensional finite difference time domain (FDTD) method to optimize the geometric parameters of the antenna. For the example shown in Figure 3b,c, the period of each unit structure is $P_{x}\times P_{y}=700\;\mu m\times 700$ μm, the unit structure is designed as an elliptic cylinder, the operating frequency is assumed to be *f_0_* = 0.228 THz, and the height of the elliptic cylinder is 480 μm. To construct the antennas which function as a quarter-wave plate and function as a dual-band perfect absorber at a frequency ranging from 0.1 to 0.25 THz, the major and minor axes of elliptic antennas should be optimized first.

**Figure 4:**
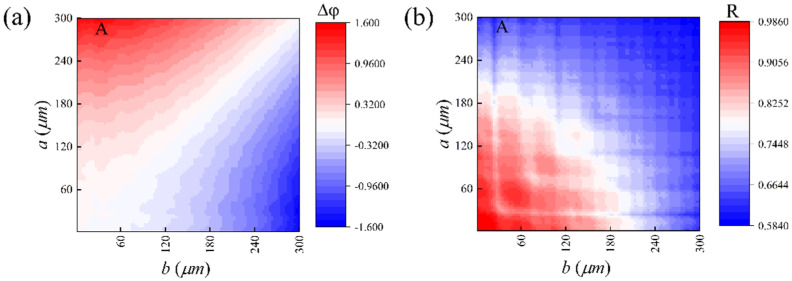
(**a**) The phase difference and (**b**) the reflectance of each antenna structure for different lengths of the major and minor axes. The temperature is *T* = 300 K, and the operation frequency is *f_0_* = 0.228 THz.

In Figure 4a,b, we obtained the phase difference by changing the lengths of major and minor axes, i.e., the difference between the phase shift of a light linearly polarized along the major axis ($\varphi_{x}$) and that along the minor axis ($\varphi_{y}$):

(6) $$\Delta \varphi =\Delta \varphi_{x}-\Delta \varphi_{y}$$

In the simulation, the spatial mesh grids are set as Δx=Δy=Δz=1 μm. The boundary condition along the *z* axis is the perfectly matched layer (PML), and the periodic boundary condition is used along the *x* and *y* axis.

**Figure 5:**
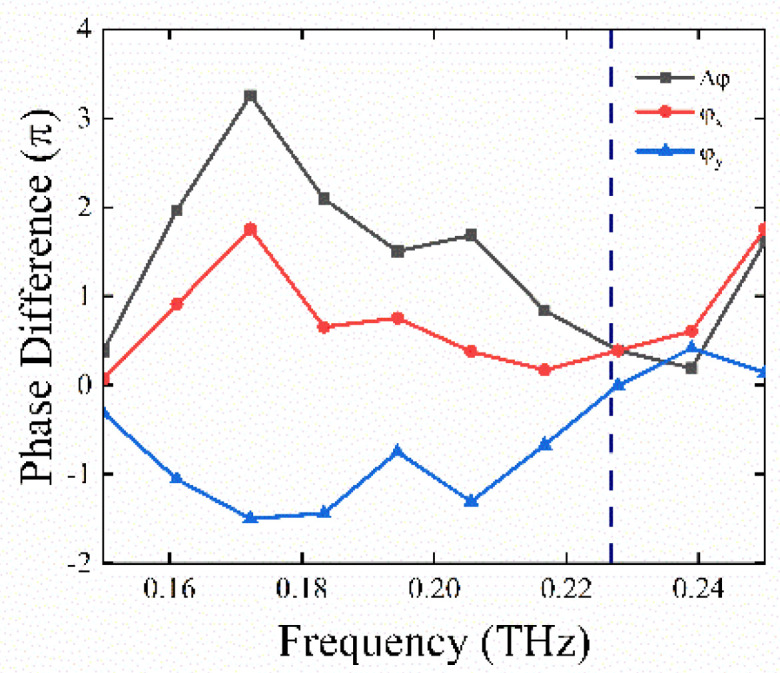
The simulated phase difference for U at a frequency ranging from 0.15 − 0.25 THz. The temperature is *T* = 300 K.

By reading the data shown in Figure 4a,b, we found that, at the point A (a=290 μm,b=48 μm,h=480 μm, the corresponding cell structure is defined as U), the phase difference and the reflectance approximate well to π/2 and 0.7234, respectively. In addition, in Figure 5, we illustrate the phase difference for U at a broadband spectrum of 0.15−0.25 THz. The results further prove that the antenna structure can be regarded as quarter-wave plates for the operating frequency.

**Figure 6:**
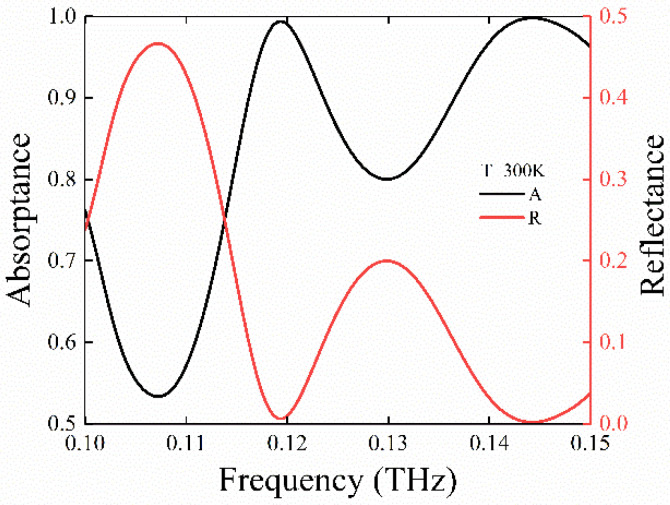
The simulated absorptance and reflectance for *U* at a frequency ranging from 0.1 to 0.15 THz. The temperature is *T* = 300 K.

Furthermore, the simulated reflectance and absorptance spectrum for U is obtained at the frequency range from 0.1 to 0.15 THz via the three-dimensional FDTD method. The absorptance is calculated by A(ω)=1−R(ω)−T(ω), where R(ω) represents the reflectance coefficient *R*, and *T*(*ω*) is the transmittance value that is equal to 0. From Figure 6, the numerical simulation result indicates that the reflectance rate of U under room temperature *T* = 300 K is decreased to 0.63% and 0.21% at 0.119 and 0.144 THz, and the corresponding absorptance is up to 99.37% and 99.79%, respectively. Obviously, from these results, we can acknowledge that U can realize polarization conversion and the perfect absorption function at a broadband spectrum of 0.1–0.25 THz under the room temperature *T* = 300 K.

**Figure 7:**
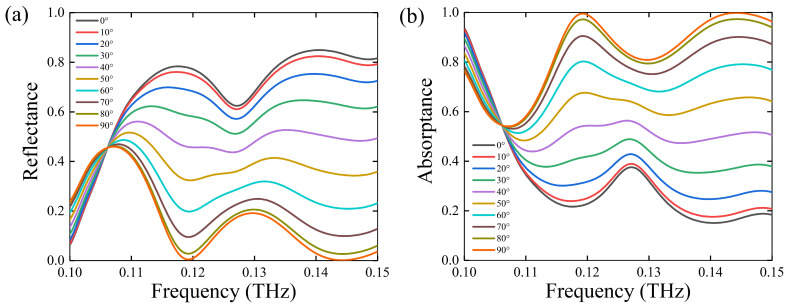
(**a**) The simulated reflectance spectrum and (**b**) absorptance spectrum for U at the frequency ranging from 0.1 to 0.15 THz when the polarization angle changes from 0° to 90°. The temperature is *T* = 300 K.

**Figure 8:**
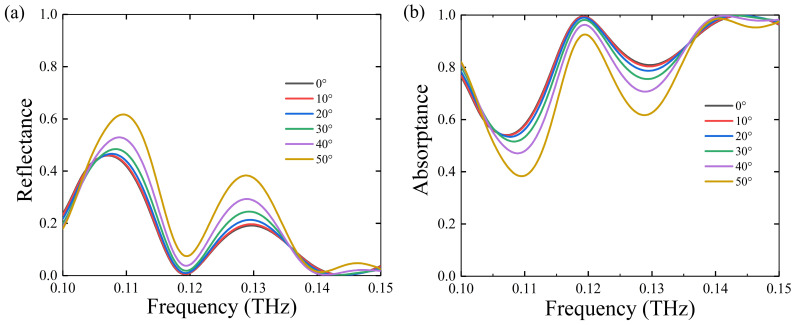
(**a**) The simulated reflectance spectrum and (**b**) absorptance spectrum for U at the frequency ranging from 0.1 to 0.15 THz when the incidence angle changes from 0° to 50°. The temperature is *T* = 300 K.

Then, we also analyzed the variation of the absorptance and reflectance spectrum under different polarization and incidence angles at *T* = 300 K, as shown in Figure 7 and Figure 8. From Figure 7a,b, we can see that the absorptance becomes lower and lower at resonance frequency with the decreasing polarization angle, which means that it is sensitive to the polarization state of the incident wave. Since there is a phase difference between the major and minor axes, it means that the absorption effect is related to the polarization; of course, we can also assume that this is caused by the shape of the sub-element structure. However, the absorptance can also reach more than 90% when the incidence angle is 0–50 degrees, as shown in Figure 8a,b, which indicates that the antenna has the effect of wide-angle absorption.

**Figure 9:**
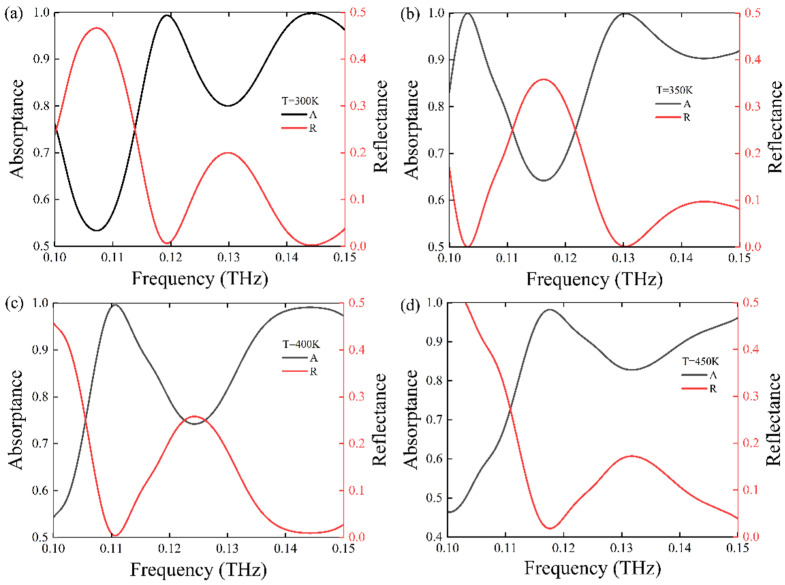
The absorptance and reflectance spectrum at a frequency ranging from 0.1 to 0.15 THz under different temperatures: (**a**) *T* = 300 K, (**b**) *T* = 350 K, (**c**) *T* = 400 K, and (**d**) *T* = 450 K.

From the analysis, we know that the permittivity of STO is sensitive to the environment temperature, which indicates that the absorbance spectra of the designed meta-surface may change with the change of temperature. When changing *T* from 300 to 450 K by a step of 50 K at a broadband frequency of 0.1–0.15 THz, we obtained the absorptance and reflectance spectrum, as shown in Figure 9a–d. From the result, we can find that the resonance frequency will shift when the environment temperature changes. Furthermore, according to the *LC* circuit model, the resonance frequency of the meta-surface can be expressed equivalently by $f_{0}\propto \frac{1}{\sqrt{LC}}$ and $\frac{1}{\sqrt{LC}}∼\frac{1}{l\sqrt{\varepsilon_{r}}}$, where *L* is the loop inductance, *C* is the capacitance, and *l* is the metallic patch length. We can acknowledge that the absorber frequency is inversely proportional to the value of $l\cdot \sqrt{\varepsilon_{r}}$; in other words, if the metallic patch length, *l,* is fixed, the absorber frequency should only be inversely proportional to the permittivity, *ε_r_*. In our theoretical analysis, *ε_r_* is equal to the Re(*ε*), so there is no doubt that *ε_r_* will be different when the environment temperature changes, resulting in a shift in the resonance frequency, as shown in Figure 9.

## 3. Results

**Figure 10:**
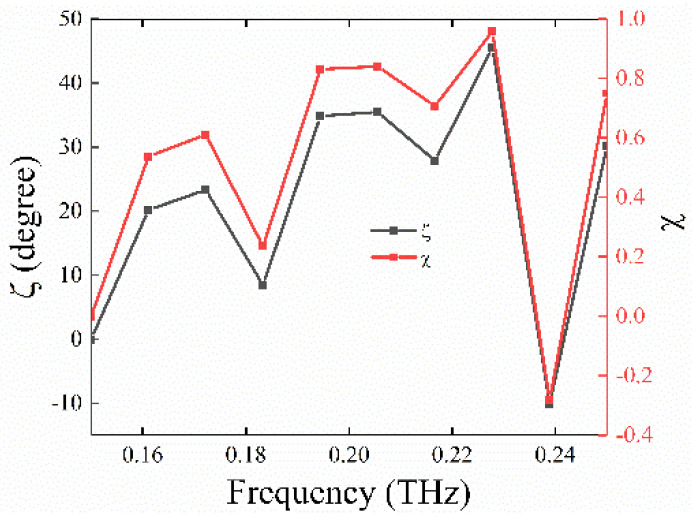
Simulated degree of circular polarization (χ) and ellipticity angle (ζ) for the incident *y*-polarized light in the range of 0.15–0.25 THz.

In order to better illustrate that the antenna structure has a perfect polarization conversion effect at *T* = 300 K, the degree of circular polarization, χ, and the ellipticity angle, ζ, from Stokes’ Formula is quoted, which can be described as $\chi =\frac{V}{I}$ and $\zeta =\frac{1}{2}\arcsin(\frac{V}{I})$ respectively, where $V=2E_{x}E_{y}\sin(\Delta \varphi )$ and $I=E_{x}^{2}+E_{y}^{2}$ [40,41]. In addition, *E_x_* and *E_y_* are amplitudes of two orthogonal vectors, and Δφ is the phase difference caused by the meta-surface. More specifically, when χ is equal to 1 or −1, it means that the measured polarized light is left or right circularly polarized light, and ζ is close to ±45°, which means that the reflection light is perfectly circularly polarized light [42,43,44]. Figure 10 shows the values of χ and ζ in the frequency range of 0.15 to 0.25 THz. From Figure 10, we can see that the value of χ is close to 1 (about 0.986), while the value of ζ is close to 45° (about 44.5°), at the frequency of 0.228 THz. The reflection coefficients of *x*- and *y*-polarized reflected waves are defined as co-polarization reflection coefficient R*_xx_* and cross-polarization reflection coefficient R*_xy_* (R*_ij_* denotes *j*-polarized reflection from *i*-polarized incidence), respectively. As is known, when the angle *θ* is equal to 90° between the light vector of incident *x*(*y*)-polarized light and the major (minor) axis of the quarter-wave plate, most of the reflection light is *x*(*y*)-polarized light. Therefore, we can see that R*_xx_* and R*_yy_* are infinitely close to 1, and R*_xy_* and R*_yx_* are infinitely close to 0 at the frequency ranging from 0.15 to 0.25 THz, while the *x*(*y*)-polarized light has passed through *U* with *θ* = 90°. This further indicates that the antenna structure has a perfect polarization conversion function under a temperature of *T* = 300 K.

**Figure 11:**
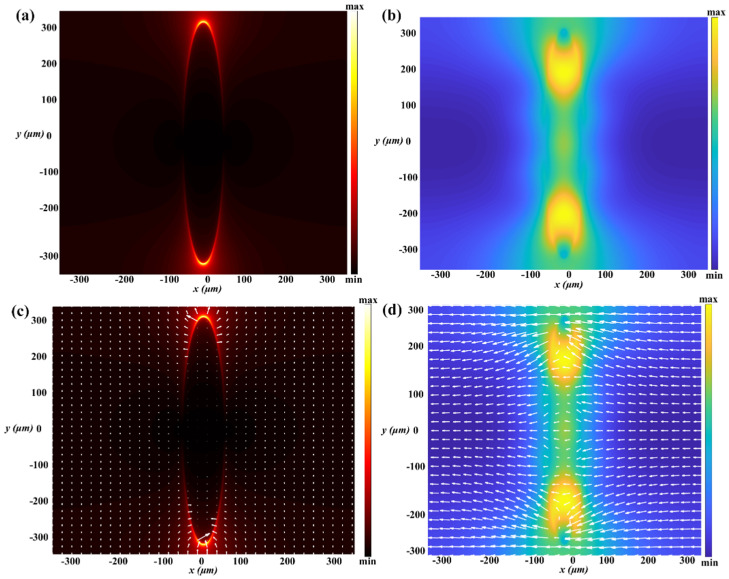
The distribution of the electromagnetic field and its vector in the x-y plane of the surface of the STO particle: (**a**,**b**) the electric and magnetic field, respectively. (**c**,**d**) The electric field and magnetic field vector, respectively. All frequencies are set at 0.119 THz and the incident light is set as *y*-polarized.

There are two types of surface plasmons: one is localized surface plasmon (LSP), which usually appears on the surface of metal nanoparticles, and the other is propagating surface plasmon polaritons (SPPs), which usually appear at the interface of metal and dielectric materials. In the case of surface plasmon resonance (SPR), we found that there are many charges and a strong field energy on the surface of the metal structure, which exhibits a strong scattering or absorption peak in the scattering spectrum; meanwhile, when the size of metal particles is less than the wavelength of incident light, it is easy to produce local surface plasmon resonance on the metal surface. We conclude that the localized surface plasmon resonance (LSPR) has occurred on the surface of the STO elliptical cylinder. The reasons for this are as follows: (1) STO material has a high refractive index in the terahertz band that has metalloid properties, (2) the operation wavelength is larger than the size of the unit structure, and (3) we have observed the galvanic couple resonance on the surface of the STO elliptical cylinder and the magnetic dipole resonance. To further understand the working mechanism while it is regarded as a perfect absorber at T = 300 K, the electric field distribution and magnetic field distribution are shown in Figure 11a,b, and the vector distribution of the electric field and magnetic field are shown in Figure 11c,d in the range of 0.1–0.15 THz. From Section 2, we know that the antenna composed of dielectric-metal particles is also regarded as a plasmon optical antenna, and when the dielectric is a high refractive index material, it is easy to cause the LSRP effect on the surface of the dielectric particle. By analyzing the distribution of the electromagnetic field on the surface of the STO elliptic cylinder, we can see that there is electric dipole resonance and magnetic dipole resonance, as shown in Figure 11a–d, which can further explain that the LSPR effect really occurred on the surface of the STO antenna. As a result, an enhanced local field is produced, which can make incidence photons effectively manacled in the sub-wavelength region of the STO antenna surface.

## 4. Prospects for Future Work

Our group proposed a new optical antenna array that can realize the polarization conversion and perfect absorption in a special frequency range without adjusting the environment temperature, changing the structural parameters, or adding active devices. When the external temperature is changed, the proposed antenna array can work well as a temperature-controlled tunable absorber. This work proves that the realization of multifunctional metamaterials does not need the complex structure or too many additional external factors. In the future, we will attempt to design the multifunctional metamaterials with richer functions and simpler structures; of course, the functions are not limited to polarization conversion or perfect absorption.

## 5. Experimental Feasibility

**Figure 12:**
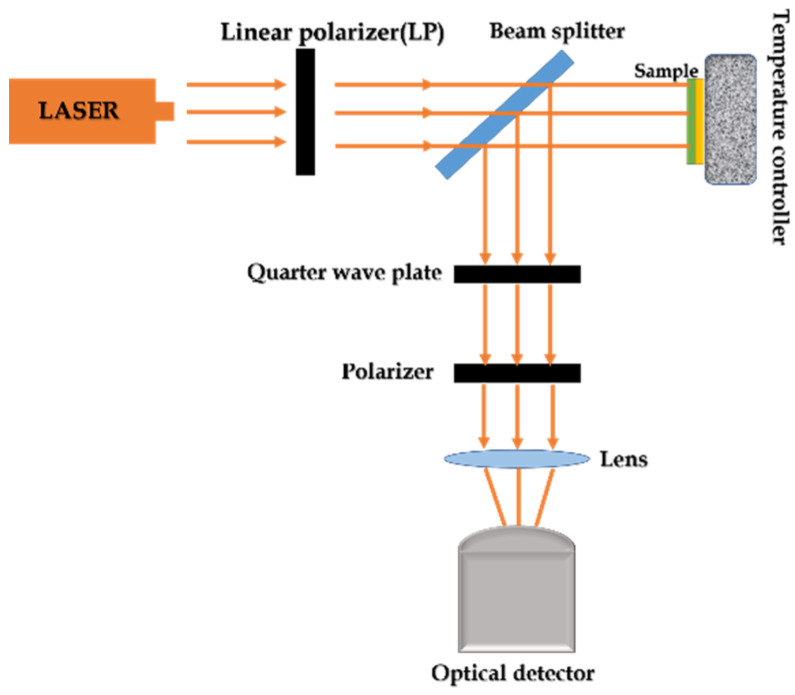
Schematic of the experimental device.

We have proposed an experimental measurement scheme, and the experiment devices is shown in Figure 12. The meta-surface was placed on the temperature controller; in this case, the reflectance and absorptance of the meta-surface at different temperatures could be measured via the optical detector. In another case, we could rotate the polarizer for one cycle, and if there are two extinction phenomena observed by the optical detector, it can indicate that the polarization state of reflected light is circularly polarized. Thus, it can be tested whether this proposed meta-surface has the functions of perfect absorption and polarization conversion.

## 6. Conclusions

Based on STO material, we have proposed and designed a single-layer multifunctional meta-surface composed of a dielectric-metal hybrid antenna array. It can realize multiple functions without adding active devices, adjusting the temperature, or changing the structure. In particular, the meta-surface can achieve the functions of perfect absorption and polarization conversion when the temperature is constant (*T* = 300 K). In addition, we have discussed the relationship between temperature and absorption when the temperature is changeable from 300 to 450 K. All working frequencies were set in the range from 0.1 to 0.25 THz. Numerical simulation results showed that, in the state of perfect absorption, the maximum absorptance can reach up to 99.97%, and in the state of polarization conversion, the degree of circular polarization and the ellipticity angle can reach 0.986 and 44.5°, respectively. The single-layer optical antenna revealed the practicability to configure a multifunctional meta-surface without adding active devices, adjusting the temperature, or changing the structure, enabling novel optical applications in the complex electromagnetic environment, such as in optical sensing, imaging, and energy harvesting.
